# Effectiveness of Phage-Based Inhibition of *Listeria monocytogenes* in Food Products and Food Processing Environments

**DOI:** 10.3390/microorganisms8111764

**Published:** 2020-11-10

**Authors:** Iwona Kawacka, Agnieszka Olejnik-Schmidt, Marcin Schmidt, Anna Sip

**Affiliations:** Department of Food Biotechnology and Microbiology, Poznan University of Life Sciences, Wojska Polskiego 48, PL-60-627 Poznan, Poland; iwona.kawacka@up.poznan.pl (I.K.); marcin.schmidt@up.poznan.pl (M.S.); anna.sip@up.poznan.pl (A.S.)

**Keywords:** Bacteriophages, biopreservation, *Listeria monocytogenes*, food quality, safety

## Abstract

Providing safe products and compliance of legal requirements is still a great challenge for food manufacturers regarding microbiological safety, especially in the context of *Listeria monocytogenes* food contamination. *L. monocytogenes* is a human pathogen, which, due to the ability of survival and proliferation in preservation conditions such as high salinity, acidity and refrigeration temperatures, is a significant threat to the food industry. Novel methods of elimination of the bacterial pathogen in food products and food processing environments are required. Among emerging technologies, one of the very promising solutions is using bacteriophages as natural control agents. This review focus on the major aspects of phage-based inhibition of *L. monocytogenes* in aspects of food safety. We describe an overview of foods and technological factors influencing the efficacy of phage use in biocontrol of *L. monocytogenes*. The most noteworthy are food matrix properties, phage concentration and stability, the time of phage application and product storage temperature. The combined methods, phage immobilization (active packing), pathogen resistance to phages and legislation aspects of antilisterial bacteriophage use in the food industry are also discussed.

## 1. Introduction

*Listeria monocytogenes* is rod-shaped Gram-positive, catalase positive, oxidase negative and facultative anaerobic bacteria [1,2]. It is naturally present in the environment and is found in locations such as soil, water, sewage, silage, plants and animals [2,3]. This organism grows at temperatures ranging from 1 to 45 °C [2,3] it also tolerates salt concentrations up to 10% and pH of 3.6–9.5 [2]. *L. monocytogenes* is an opportunistic pathogen of humans, causative of an illness named listeriosis [1,2,4,5]. Traditionally, the division of *L. monocytogenes* into serovars is based on the unique combination of their surface proteins: somatic (O) and flagellar (H) antigens. There are 14 *L. monocytogenes* serovars [6,7,8] and out of them serotypes 1/2a, 1/2b and 4b are causative factors of the majority (some sources declares 95–98%) of human listeriosis cases [6,9,10].

Ingestion of highly contaminated foods in most individuals leads to mild to severe gastroenteritis [11] with symptoms of the illness including diarrhea, abdominal pain, fever, vomiting, mild and flu-like illness [2]. Pregnant women, neonates, elderly and those who are immunocompromised are groups with the highest risk of becoming infected with *L. monocytogenes* [1,4]. In the case of those groups, consumption of food contaminated even at low levels can cause sepsis, subsequent bacterial meningitis, and/or infection of the fetus, leading to spontaneous abortion, stillbirth or premature birth [2,11]. The mortality rate of listeriosis reaches 20–30% [2,4].

Due to the ability of survival and proliferation in food preservation conditions such as high salinity, acidity and refrigeration temperatures *Listeria monocytogenes* is a serious threat to the food industry [2], as this pathogen intermittently can be found in almost all raw food materials [3]. Unprocessed ingredients (e.g., raw meat and fish, unpasteurized milk or uncooked, fresh vegetables) are sources of contamination in final food products but food can also be contaminated after processing [2]. Various contaminated food products, including vegetables, milk and dairy, red meat, poultry, seafood and diverse ready-to-eat (RTE) products, such as salads and smoked fish products were sources of listeriosis infections [2].

*Listeria monocytogenes* has the ability to form a biofilm, where bacteria anchor themselves with extracellular polymers to surfaces (including polystyrene, stainless steel and Teflon) [12,13,14,15]. Bacteria in biofilm are partially protected from chemical cleaners and disinfectants [12,14], which make them especially difficult to remove from food processing environment, machines and equipment, in some cases leading to contamination of the final product [13].

Listeriosis outbreaks are a worldwide problem with reported cases from Europe, Australia, New Zealand, Asia, Africa, USA, Canada and South America [1,2,16,17].

Regulations regarding the presence of *L. monocytogenes* in food depends on local authority rules. In the USA there is a so-called “zero-tolerance policy” [18]. The regulations require the absence of *L. monocytogenes* in 5 × 25 g of food and also in the food-processing environment. Such requirements pose a serious challenge to food manufacturer and enterprise that is exporting to the United States [9,19]. European Union legislations require absence (10 × 25 g samples) of *L. monocytogenes* in food intended for newborns and special medical purposes, in RTE food products with the ability to support growth the pathogen *L. monocytogenes* must be absent in 5 × 25 g at the time product leaves the manufacturer or it may not exceed 100 CFU/1 g throughout its shelf live and in RTE products not supporting the growth of *L. monocytogenes* the numbers should not exceed 100 CFU/1 g of product throughout the whole shelf-live [9,19]. Similar regulations are applied in Australia/New Zealand and Canada [9,19].

Because providing safe food products and compliance of legal requirements are still a great challenge for food manufacturers, novel methods of eliminations of pathogens (including *L. monocytogenes*) in food products are required. One of the promising solutions is using bacteriophages as novel control agents [9,20].

## 2. Bacteriophages

Bacteriophages (phages) are deemed the most abundant biological entities in the biosphere [14,20,21]. These are viruses that infect bacteria that can be found in every environment, where host bacteria live, including water, fresh foods and they are also present in the human gut [12,14,22,23]. It is estimated that over 10^31^ various phage particles can be found on Earth [20,21,24]. They are highly specific targeting only a narrow range of permissive bacteria [24]. Their host specificity is usually found at a subgroup of strains (mainly within the same species or across very closely related species) [22]. The phage discovery by Frederick W. Twort and Félix d’Hérelle over 100 years ago was unsurprisingly related to the idea of phage therapy. The leading research centers in the field of phage therapy were from the former USSR and Poland. The major scientific groups which continue their research in the area since the beginning of the 20th century are located in The Eliava Institute of Bacteriophage, Microbiology and Virology (Tbilisi, Georgia) [25] and The Hirszfeld Institute of Immunology and Experimental Therapy (Wrocław, Poland) [26], despite the triumph of antibiotics in 1940s [27]. All available evidence indicates that phages are harmless to humans, including their consumption [24]. For example in a repeated dose toxicology study in rats it was found that purified *Listeria monocytogenes* phage P100 preparation is safe and well-tolerated. No mortality or morbidity related to P100 were observed and also histopathological examination did not reveal any changes [28].

Phages, depending on their replication cycles, can be categorized into two groups: lytic (or strictly lytic, also called virulent) and temperate (or lysogenic) [15,20,29,30,31]. Lytic phages after entry to the host cell initiate the process of producing multiple viral progenies subsequently released from the host cell due to accumulation phage lytic enzymes [20,31], causing death of the host cell [24,29,30]. Temperate phages have an ability to perform a lytic cycle but also alternatively a lysogenic cycle. In the latter one the prophage is formed by integration a genetic material of the phage into the host genome (also circular or linear plasmid can be formed) [20,30]. Prophage replicates with the host genome usually in quiescent state until the lytic cycle is induced [20].

Phages have many applications in healthcare, veterinary and agricultural sectors for phage-based detection of pathogens, biocontrol (inhibition of pathogens in foods) and as therapeutics [24,32]. Phage therapy of listeriosis is not possible, because the bacteria invade host cells and proliferate or persist as intracellular pathogens. In that state it cannot be reached by phages or even antibiotics [24].

### Bacteriophages-Based Inhibition of Pathogens in Food Products

Bacteriophages are considered a great weapon against pathogens in food products because of their properties: not influencing microflora other than targeted (what is especially useful in fermented products) [12,23,33] and not influencing the sensory properties of the final product [12,33]. For example, in performed study *Listeria monocytogenes* phage P100 did not appear to interfere with the functional lactic acid bacteria in the cheese or to change the characteristics of the product [34]. The commercially available product, ListShield™ (containing a mix of *Listeria monocytogenes* phages), did not impact the organoleptic properties (including taste, sight and smell) of pre-sliced oven-roasted turkey breast, cooked ham, meat bologna and roast beef [22].

Because of these properties phages are used in food processing plants to target many pathogens such as *Listeria monocytogenes*, *Salmonella* serotypes, *Escherichia coli* 0157:H7, *Shigella*, *Clostridium perfringens* and other [20,21,23]. There are more than 25 commercially available bacteriophage products produced under 6 brand names [21,23]. Commercial phage biocontrol products are usually water-based phage suspension with low level of salts and free of any additives or preservatives [33].

An ideal phage applied to food processing or food products directly should meet specific requirements [24,28,35,36]: strict virulence (phage infection should result in host death but not in lysogenic conversion of its host); broad host range for infecting and killing as many target strains as possible; an inability for performing generalized transduction, as some phages may transfer genetic material, including virulence factors (especially toxin genes) from one host to another; absence of genes encoding pathogenicity associated or potentially allergenic proteins, as well as oral feeding studies should not indicate any adverse effects; sufficient stability over storage and application; propagation in a non-pathogenic host (to avoid processing large-scale pathogen cultures); ability to scale up for commercial productions.

Even though phages are a potential weapon to treat pathogens already used in some countries, there is a concern that widespread use of these bacteriophage treatments may eventually lead to the emergence and/or selection of phage resistant mutants [33,37,38]. Minimizing chances for diminishing of phage treatment efficacy due to phage resistance, several measures can be implemented. Phage-treated products should not re-enter the production cycle. This can be achieved by phage administration to products immediately before packaging and/or shipment to avoid de novo contamination and/or emergence of a phage-resistant bacteria in a production environment. Strict avoidance of specific working routines like recycling inoculation loops (e.g., in so-called “young-old smearing,” where the rind microflora from mature cheeses is used to wash young cheeses). Biocontrol products based on virulent phages with a broad host range are used, as well as mixture/cocktail products containing more than one phage with different host specifities, preferably in rotating application schemes [33,39,40].

Finally, in case resistance does arise, phage cocktails could be modified to contain additional phages specific to resistant bacteria [33]. Selection of new phages targeting phage-resistant bacteria is a relatively rapid process [31]. Moreover, in case of bacteriophages (unlike e.g., antibiotics), a process of antagonistic coevolution may happen. In such instances occurrence of the host tending to be more resistant and the phage tending to have broader host range is expected [29]. For example, phages can adapt by expressing adhesion proteins targeting new receptors, thus overcoming bacterial resistance [13].

It is worth mentioning that an alternative method of indirect usage of bacteriophages for the biocontrol of pathogen growth, is using their endolysins (lysins), without using viable phages directly in foods [12]. Endolysins are hydrolytic enzymes, synthesized at the end of the lytic cycle in order to allow the release of the phage progeny by bacterial cell wall hydrolysis. This process is termed lysis from within [12,14] but suspensions of endolysins are also able to lyse the cell from the outside (in a process called lysis from without) [12]. There are many publications describing characterizations of new endolysins with activity against foodborne pathogens and spoilage bacteria such as *Staphylococcus aureus*, *Clostridium tyrobutyricum*, *C. sporogenes*, *C. perfringens*, *Bacillus cereus*, *Listeria monocytogenes*, *Pseudomonas aeruginosa* and others [41].

## 3. Listeria Monocytogenes Bacteriophages

In total there are more than 500 phages specifically infecting *Listeria* sp. that have been identified, however, the majority of known phages are temperate [35,36] so most of them are not very useful for inhibition of *Listeria monocytogenes* in food products and food processing plants.

Lytic bacteriophages however are used as a tool for biocontrol of *Listeria monocytogenes*. There are two commercially available phage-based products targeting *L. monocytogenes* in food products and food processing environments. ListShield™ (formerly LMP-102) is produced by Intralytix Inc (Baltimore, MD, USA) and PhageGuard Listex™ (formerly Listex™; P100) is produced by Micreos Food Safety (formerly EBI Food Safety) (Wageningen, Netherlands); [21,33,42]. ListShield™ is a cocktail of 6 distinct lytic phages: LIST-36 (ATCC # PTA-5376), LMSP-25 (ATCC # PTA-8353), LMTA-34 (ATCC # PTA-8354), LMTA-57 (ATCC # PTA-8355), LMTA-94 (ATCC # PTA-8356), LMTA-148 (ATCC # PTA-8357) [13,22,43], while PhageGuard Listex™ consists of only one broad host range phage P100 [44].

Properties of particular phages are different and obtained results in the pathogen inhibition depends on the phage choice. For example, P100 is closely related to *Listeria* phage A511. Both phage species express a broad (but nevertheless different) host range within the genus *Listeria* and belong to the same morphotype family (*Myoviridae*) [28]. It was showed that both viruses have very similar efficacies against *L. monocytogenes* 1001 [39]. P100 was shown to be less heat-resistant than A511 and fully inactivated after 30 s at 71 °C. However, it was able to regain activity even after heating to 80 °C. Phage A511 was less sensitive to heat (not inactivated by exposure to 71 °C) but was not reconstituting after incubation at 80 °C [45].

Phage P70 on the other hand was found to show a remarkably broad host range infecting with similar efficiencies *Listeria* sp. serovars: 1/2a, 1/2b, 1/2c, 4a, 4c, 4d, 4e, 5, 6a and 6b. Over 62% of *Listeria* strains tested (*n* = 29) were lysed by P70. The tested strains included food isolates and reference strains. P70 has a distinct virion morphology, genome size and structure unrelated to any other *Listeria* phage described so far [46]. Another example of *Listeria* lytic phage, which was assigned to a *Homburgvirus* genus, is LP-018. It possesses the distinct property being able to infect phage-resistant mutants characterized by loss of rhamnose moiety in teichoic acids (a common receptor within bacterial cell wall) [47].

### 3.1. Phage Based Biocontrol of Listeria Monocytogenes in Foods

There were many attempts to use bacteriophages for biocontrol of *Listeria monocytogenes* in food products, including raw meat, smoked fish, fermented fish, milk, cheeses, fresh-cut fruits, vegetables and various ready to eat products. In the majority of trials, authors succeeded with the reduction or even eradication of *L. monocytogenes* from food products. Most of the trials were performed with P100 phage, often in a form of commercially available PhageGuard Listex™, several experiments were done with bacteriophage cocktail ListShield™ and only few attempts were performed with other bacteriophages. Experiments were performed with phages only or in combination with other cofactors. For detailed information see Table 1.

The first experiment performed on a model food system was done by Dykes and Moorhead [48] using bacteriophage LH7 on vacuum-packed raw beef. The presence of listeriophage alone had no significant effect on the number of *L. monocytogenes* over the control. Hagens and Loessner [24] suggested that the authors used a concentration of bacteriophage solution (3 × 10^3^ plaque forming units per milliliter (PFU/ mL)) that was too low, in which the meat was immersed. That first attempt resulted in lack of significant decrease of the pathogen with phages only (thought phages enhanced performance of nisin), however other studies showed in the Table 1 provided consistent results that using a bacteriophage or mix of bacteriophages can lead to a significant reduction of *L. monocytogenes* with appropriate phage/*Listeria* ratio under certain conditions in most food products.

For example, Leverentz et al. [50] optimized the application technique and titer of phage suspension on honeydew melons and enabled a reduction of *L. monocytogenes* up to 6.8 log units during storage for 7 days. Bigot et al. [52] in their experiment on phage application to the surface of vacuum-packed ready-to-eat chicken breast roll, obtained an immediate 2.5 log CFU/cm reduction of viable pathogen contamination and the re-growth was prevented over 21 days incubation in 5 °C. Hong [58] found that phage mix LMP-102 reduced populations by one log compared to the control *L. monocytogenes* counts on honeydew melon pieces from 2 to 7 days. Iacumin et al. [60] obtained eradication in certain conditions (with low initial *Listeria* titration and high phage titration) and Sadekuzzaman et al. [13] and showed a reduction of biofilm on lettuce leaves. Figueiredo and Almeida [64] stated that phage P100 decreased *L. monocytogenes* to undetectable levels at zero and 72 h post-infection (6–8 °C) on sliced ham and also Yang et al. [43] obtained a significant reduction of the pathogen on chicken breasts.

There are many factors that influence phages’ effectiveness. Prediction of the phages activity in a complex food matrix is very difficult. Interaction with phages and their hosts can be influenced by many factors, such as ionic strength, pH, food form (e.g., liquid or solid) and presence of other substances which may interfere with phage particles diffusion, receptor recognition and binding, cell wall and membrane penetration, as well as virus genome internalization processes [39]. Factors influencing the effectiveness and their impact of phage treatment are described below.

#### 3.1.1. Food Form

The food matrix or in other words the form of food product has an impact on bacteriophage treatment effectiveness.

Guenther et al. [39] suggested that phages suspended in liquid foods can diffuse almost freely and thus their distribution and potential contact with their host cells does not appear to be a problem. While, on solid foods such as hot dogs, salad leaves and so forth that have an even surface, where the surface properties (e.g., liquid adsorption ability) or total surface area accessibility are limited the parameters may be of great importance. According to the authors, the most challenging foods to treat with phages are those with an uneven and large surface area (fish, meat and seafood), because phage particles have limitations to reach all bacterial targets. Moreover, the pathogens may penetrate into the food matrices, thereby hiding from diffusing phage particles. Oliviera et al. [57] found that the effectiveness of reduction *L. monocytogenes* with P100 phage in fruit juices was higher than on the fruit slices, which is consistent with previous findings.

#### 3.1.2. Phage Concentration

Results provided by many authors are consistent that in general, increasing the initial phage/*Listeria* ratio (either by reducing *L. monocytogenes* titration or by increasing phage titration) enhanced the efficacy of the phage in reducing bacterial populations [28,39,44,49,50,52,60,70]. Apart from tested food models, under real conditions in food products using a sufficiently high titer of phage suspension at the beginning of the treatment, without relying on self-amplification is crucial. Mainly, because of the usually low number of target cells present in foods, which limit replication and a further increase of phage concentration [39].

Exact phage titration leading to a significant decrease of *L. monocytogenes* depends on many factors, such as a method of application, counts of the pathogen and the food itself. For example, tests performed by Leverentz et al. [50] demonstrated that phage concentrations of 10^6^ through 10^8^ applied with a spray gun on honeydew melon tissue caused suppression of pathogen growth throughout the storage period of 7 days at 10 °C. Carlton et al. [28] observed complete eradication of viable *L. monocytogenes* on the soft cheese model when the concentration of 3 × 10^9^ phages per ml of smearing solution that was used. The results of Soni et al. [44] showed that P100 phage at a dose of 2 × 10^7^ PFU/g was necessary to significantly reduce *L. monocytogenes* counts on fresh catfish fillet.

The concentration of phage in the suspension used to apply on treated food must have high enough titer to ensure the contact of the passively diffusing virus particles with their host cells, within a given time and considering spatial limitations of food matrix [39].

#### 3.1.3. Product pH

The data from several studies are in agreement that the application of the phages on apple slices is much less effective than on the melon slices [49,50,57]. It is suggested that lower pH of apple tissue (compared to melon) may be the reason for the issue. For example, in the experiment performed by Leverentz et al. [49] the phage cocktail reduced *L. monocytogenes* populations by 2.0 to 4.6 log units over the control on honeydew melons (pH was approximately 5.5 to 6.5), when on apples (with pH 3.8 to 4.2), the decrease in the pathogen cells number was below 0.4 log units. The same experiment indicated, the activity of phage particles was stable on melon slices but declined rapidly on apple slices. In the similar experiment performed by Oliviera et al. [57] phage treatment with P100 was more effective on fresh-cut melon then on pears. Observed decrease in *Listeria* count was, respectively, about 1.50 and 1.00 log CFU/plug) but again the phage applied on apple slices was not effective. In the same paper, similar results regarding fruit variety were obtained regarding reduction of *L. monocytogenes* in juices. The number of the pathogen cells in apple juice remained unchanged after phage treatment. The authors attributed inefficient *Listeria* removal in the apple juice (pH 3.70) to the increased sensitivity of phages to the acidic environment which resulted in a marked decline of phage activity. However in other study, phage P100 was tested in TBS media and authors did not observe a significant reduction in phage P100 titer over the pH range 4–10 within 24 h, what indicates that P100 is stable over a wide pH range. Authors observed that P100 titer rapidly decreased by more than 5 log units within 1 h, only when the pH values were equal or lower than 2 and equal or higher than 12 [71]. Leverentz et al. [50] provided a possible explanation of these issues after their experiments, where the phage cocktail retained its activity in the low pH of 3.8 in the presence of MnCl_2_. Authors suggested that phages may lose their ability to interact with receptor or attach to the host cell surface or infect their bacterial hosts by a specific acid or some other compound present in the apples rather than the decline in effectiveness is solely a pH effect.

Nevertheless, Oliviera et al. [57] suggested that the efficacy of a low pH product treatment with bacteriophages could be improved by application of higher titer of phage or the development of low-pH tolerant phage mutants. Other alternatives mentioned involved combination of phage cocktail with other preservatives, such as bacteriocins, antagonistic bacteria and essential oils.

#### 3.1.4. Stability of the Phage

Phage stability depends on the food matrix on which it is applied. On food of animal origin phages usually remained stable, for example, phage P100 remained stable on the raw salmon fillet tissue over a 10-day storage period [55], the same phage was stable on catfish fillet samples [44] and the homogenized cheese surfaces and no significant decrease or increase in phage titer were determined over a period of 6 days [28]. Other experiments showed consistent results on queso fresco cheese, hake fillet tissue, dry-cured ham slices and other animal-origin products [39,42,59,60]. In contrast, the incubation of viruses with cabbage and lettuce resulted in a more-significant reduction of infective particles up to 2.0 logs at 20 °C [39]. However, other findings showed that P100 was stable over 10 days period on Coleslaw [66]. Moreover as discussed above, the phage titer was stable on melon slices but declined rapidly on apple slices [49].

#### 3.1.5. The TIME of phage Application

Studies show that the time of application of the phages is also an important factor. Leverentz et al. [49] performed an experiment where a phage cocktail was applied to honeydew melon pieces at 1, 0.5 and 0 h before contamination with *Listeria monocytogenes* and 0.5, 1, 2 and 4 h after contamination. The phage treatment was most successful when exert not earlier than 1 h before contamination. This suggests that phages need to be used during or directly after slicing the product to be effective against potential contamination that may occur before packaging.

#### 3.1.6. Temperature of Storage

Conditions of storage after phage application, including temperature, may also significantly affect plaquing efficiencies [72,73]. In general higher temperatures of post-phage treatment, storage leads to greater percentage reduction of the *L. monocytogenes* compared to phage-untreated control, however, due to shorter generation time of the pathogen final count of *L. monocytogenes* is also greater.

For example contaminated hot dogs, chocolate milk and cabbage were treated with phage A511 and stored at 6 °C or 20 °C. The percent reductions were greater at a higher temperature, however, the viable counts of *Listeria* in the phage-treated samples were also higher at 20 °C [39]. On contaminated dry-cured ham slices treated with P100 stored at 4 °C, 10 °C or 20 °C the count of survived *L. monocytogenes* increased with the increasing temperature, hence the lowest counts of the pathogen were obtained at 4 °C [60]. The difference between counts on treated and untreated cheese also varied considerably by temperature–the smallest difference between phage-treated and untreated cheese on the first day of the experiment was found for cheese incubated at 6 °C, with higher corresponding differences at 14 °C and 22 °C [67]. In sashimi at 3 °C no significant *L. monocytogenes* reductions were observed with the P100 treatment (on any concentration) at 2 log/g *L. monocytogenes* initial contamination for most of the measures. However, with the same initial *L. monocytogenes* concentration, the most effective (statistically significant) treatment was obtained with P100 added with the highest concentration (8 log CFU/g) at 22 °C [63].

#### 3.1.7. Regrowth of *L. monocytogenes* in Phage-Treated Food Products

Even if a great reduction of *L. monocytogenes* after application of phages is observed, some of the authors observe regrowth during further storage. A study performed by Rossi et al. [53] indicated that P100 is inhibitory against *L. monocytogenes*, in the samples of the Brazilian fresh sausages under refrigeration (4 °C) for 10 days, although the counts of the viable cells of the microorganism had increased over the storage period in comparison with day 1. Similar results were obtained by Soni et al. [42], where treatment queso fresco cheese with P100 decreased *L. monocytogenes* counts by 3–5 logs after 24 h but were unable to prevent *L. monocytogenes* growth in subsequent refrigerated storage. Regrowth of the pathogen was also observed by others on many food types [50,54,56,67,68,69]. The problem may be a result of possible overestimation of the phage killing effect that may occur in some performed experiments. The source of this error may be a lack of the phage inactivation or removal before seeding assays, resulting in possible contact of bacteria with the remained phages [74]. To overcome this source of error, Chibeu and Balamurugan [74] provided the protocol for assessing the effectiveness of tea extract based virucide application to remove phage LISTEX™ P100 not bound to *Listeria monocytogenes* from stomached rinses with tea infusion as a virucidal agent.

#### 3.1.8. Combined Treatment

There were many experiments with more than one factor intended to prevent *L. monocytogenes* growth added to the food matrix. Most of the performed studies resulted in a greater reduction of the pathogen either by additive or synergistic effects. Tests were performed with bacteriocins, chemical preservatives, protective cultures, UV light and High Pressure Processing (HPP).

The factor used the most often for the experiments with combination treatment were bacteriocins and among them, nisin was the most frequently tested. Some of the authors obtained a greater reduction of *L. monocytogenes* with combinations of bacteriophages and nisin than with using these factors separately [48,49,55].

Dykes and Moorhead [48] in their experiment used mixed treatment of bacteriophage LH7 and nisin. The combination did not prevent the regrowth of the *L. monocytogenes* strains incubated at 30 °C but in the fridge simulated conditions (7 °C) regrowth was not observed for extended periods or at all. Soni et al. [55] suggested that enhanced efficacy of phages combined with nisin could result from the pore formation by the bacteriocin, which subsequently provides extra channels for phages (phage P100 in case of their experiment) to enter *L. monocytogenes* and achieve the cell lysis. However, Figueiredo and Almeida [64] obtained results showing that the combination of nisin and P100 phage had a small antilisterial effect right after treatment, indicating rather antagonistic relation between these agents. At 72 h, almost 3 log cycles of reduction were observed in the number of viable bacterial cells compared to the untreated control, the results however were still statistically significantly worse than treatment with phage alone. In the experiment performed by Lewis et al. [66] on the other hand, P100 and nisin in combination were not more effective than P100 alone–obtained reduction of *Listeria* was greater in combination but the difference was not statistically significant.

Baños et al. [59] used enterocin AS-48 accompanying phage P100 and this combination reduced *Listeria* below detection levels from 1 to 15 days, which was more effective than using these components alone. Komora et al. [69] observed synergism between phage P100 and pediocin PA-1 for one strain of *Listeria monocytogenes* in milk samples at three and seven days of storage, for another strain synergism was only observed immediately after treatment.

Holck and Berg [38] and Hong [58] used protective cultures as a cofactor. First authors used *Lactobacillus sakei* TH1with Listex™ on slices of cooked ham, whereas Hong [58] used *Gluconobacter asaii* together with ListShield™ on honeydew melon slices. Both studies resulted in a greater reduction with the protective culture than with phages alone.

Another alternative for pathogen control is using preservatives. Their interactions with phages have also been tested. Chibeu et al. [54] obtained a greater reduction of the pathogen on cooked turkey treated with phage and potassium lactate and on the roast beef treated with phage and potassium lactate and sodium diacetate mix at 4 °C over 28 days compared to products treated with phage only. These findings are consistent with results by Soni et al. [42], where potassium lactate and sodium diacetate mix together with the phage resulted in a greater effect than using phage only on Queso fresco cheese. In the same paper using lauric arginate (LAE) in addition to phages also provided better control over *L. monocytogenes*, however in the later paper regarding cold-smoked salmon Soni et al. [55] observed that phage and LAE resulted in worst effect than phage alone (but there was no statistical significance). Oladunjoye et al. [65] in their study used a combination of phage P100 with sucrose monolaurate on carrot and tomato fresh produce, which resulted in higher log reductions on both fresh produce when sucrose monolaurate was used at 400 ppm.

Alternate alternative factors tested were UV-C light (on chicken breasts) and high-pressure processing (in milk). In both experiments using another cofactor provided better results than using phages alone [43,69].

All information provided suggest that phages can be a part of complex antimicrobial treatment to prevent *L. monocytogenes* growth in food products, providing desired natural products (see Table 1).

### 3.2. Phages Immobilization–Antilisterial Active Packaging

Immobilization of listeriophages emerged as an alternative method of its application to foods in a form of active packages.

Due to the charge difference within the external phage structure, phages can be immobilized on surfaces through electrostatic interactions [75]. Modified cellulose membranes were used to phage immobilization with the utilization of this property. Anany et al. [75] in their experiment indicated that immobilized *Listeria* phages can be applied to RTE meats packaged under vacuum or modified atmosphere packaging (MAP) to limit the growth of *L. monocytogenes* to less than 0.5 log cycles in RTE foods throughout their shelf lives. Lone et al. [61] compared the efficacy of immobilized and free phages and controlling *L. monocytogenes* growth in contaminated cantaloupe using phage suspension applied by pipetting appeared to be more effective than application of immobilized phages. However, the titer of immobilized phages on cellulose membranes was not determined, thus their lower efficacy may be attributed to differences in the number of phages applied per unit of surface area and thereby with lower numbers of infective phage particles being present. Nevertheless, authors obtained significant reductions of the pathogen with immobilized phages compared to untreated control.

Radford et al. [67] immobilized bacteriophages to xanthan coatings applied on poly-lactic acid (PLA) films, which showed 99.99% phage release within 30 min of contact with produce. In their experiment on pre-cooked turkey breasts, populations of the pathogen cells were significantly declined on samples having contact with the xanthan immobilized phage on PLA, in comparison to the xanthan coated PLA or uncoated PLA alone. Significant inhibition of *L. monocytogenes* growth was observed in both the aerobic and the vacuum packaging at tested temperatures: 4 °C and 10 °C. *L. monocytogenes* counts increased over time under all treatments but with phage, the increase was significantly smaller in the range of 2 logs decrease of the pathogen compared to the controls. Though significant, this reduction of *L. monocytogenes* growth was 3 logs below what has been previously reported for this meat system by Chibeu et al. [54]. Taken together these findings suggest that phage application in conjunction with other antimicrobial control methods, rather than as a standalone, is an answer to *L. monocytogenes* contamination [76]. Attempts to immobilize antilisterial bacteriophages are summarized in Table 2.

### 3.3. Inhibition of Listeria Monocytogenes on Surfaces

Phages can be employed to eliminate or reduce *L. monocytogenes* biofilm on the surfaces in food processing plants. Phage-derived enzymes were proved effective in degrading the exopolysaccharide, the major constituent and protective substance of the biofilm matrix, thus phages are able to reach biofilm cells and destroy the biofilm bacteria. Moreover, it is presumed that bacteriophages are capable to diffuse through the pores and channels of biofilms to reach internal layers of the biofilm [13]. However, Chaitiemwong et al. [77] performed an experiment on stainless steel with grooves and the results indicated that only in the 0.2-mm grooves (the shallowest among tested) the phages were likely to be more effective in the biofilms with food matrix than the chemical disinfectants.

However, it was shown that *Listeria* phages (at concentrations of 3.5 × 10^8^ PFU/mL) may be as efficient as a 20 ppm solution of QUATAL on sanitizing stainless steel and polypropylene surfaces contaminated with *L. monocytogenes* [78]. The phages successfully inhibited L-form biofilm formation on stainless steel and were as successful as lactic acid (130 ppm) at inactivating pre-formed L-form biofilms [79]. The phage P100 treatment significantly reduced *L. monocytogenes* cell populations under biofilm conditions on the stainless steel coupon surface, where there was a 3.5- to 5.4-log/cm^2^ reduction in *L. monocytogenes* cells by phage treatment [80] and in other study authors obtained complete biofilm elimination on stainless steel [60]. In other experiment P100 at concentrations equal to or greater than 8 log PFU/mL successfully removed *L. monocytogenes* biofilms from polystyrene surfaces [81].

On the other hand, Ganegama Arachchi et al. [82] obtained results showing that phages possess reduced efficacy for the lysis of biofilm embedded cells compared with recently attached exponential phase cells of *L. monocytogenes*. Once displaced from the biofilm matrix, the cells were sensitive to phage treatment. However, it cannot be excluded that the increased sensitivity of cells released from biofilm matrix might be a result of biofilm disruption procedure. Therefore, their results indicate the need for other additional treatments to maximize the effect of phages on mature biofilms of *L. monocytogenes*. The summary of the attempts with reducing biofilm from surfaces is presented in Table 3.

## 4. *Listeria* Resistance to Phages

Although strategies to delay or eliminate the emergence of bacteriophage-resistant *Listeria* strains exist and were described earlier in the text, rapid emergence and/or selection of phage resistant mutants still represents a concern associated with the use of phages to control *L. monocytogenes* [37].

Not all *Listeria monocytogenes* strains are sensitive to all phages, even broad-host ones. For example, Vongkamjan et al. [84] in their work collected a total of 595 samples from raw material, finished seafood products and environmental samples from different sites of a seafood processing environments in Thailand. Out of the samples, 22 (3.7%) were positive for *L. monocytogenes* and five isolates showed resistance to 12–20 out of 29 phages. In other work, *L. monocytogenes* samples from a smoked fish processing facility, representing persistent subtypes, were characterized against a panel of 28 *Listeria* phages and this work showed a wide range of likelihood of phage susceptibility, ranging from 4.6% (for 7 ribotype DUP-1043A isolates) up to 95.4% (for 7 ribotype DUP-1044A isolates), what indicated that some of the subtypes can be resistant to the majority of phages [37].

The issue raised the question if *Listeria* strains originally sensitive to phage recovered from phage treated samples gained phage resistance. Cartlon et al. [28] performed an experiment in which none of the *Listeria* clones isolated from cheeses receiving low concentrations of P100 revealed resistance against the phage. Guenther et al. [39] also did not find any bacteria isolated from phage-treated foods to be resistant against the phages used (A511 and P100) in plating assays. Later experiments on *Listeria monocytogenes* isolates from phage treated food products brought similar results [54,61,66].

However, Fister et al. [85] in their experiment screened 486 *L. monocytogenes* isolates obtained from 59 dairies over 15 years for the presence of P100 insensitive *L. monocytogenes* strains. The overall number of P100 insensitive strains was 13 (2.7%) for the whole collection timespan and investigated plants. Interestingly, the detected insensitive strains originated from the dairy plants where phage treatments were applied indicating probable emergence of phage-resistance due to existence of the selective pressure.

What is more, Vongkamjan et al. [37] performed an experiment in which *L. monocytogenes* isolates recovered of phage cocktail treatment after 24h showed diminished susceptibility to individual phages used in the phage cocktails, indicating possible and rapid appearance of phage insensitive mutants under selective conditions. The experiment performed in laboratory model in broth at 30 °C what does not reflect conditions present in foods stored at refrigeration temperatures and in presence of accompanying bacteria.

Further studies on phage resistance mechanisms in *Listeria monocytogenes* were performed. Denes et al. [86] isolated 69 spontaneous mutants of *L. monocytogenes* strain 10403S that were resistant to phage LP-048 or LP-125 (or both). All phage-resistant mutants generated in the study acquired the insensitivity to the phage(s) based on adsorption inhibition. Of note, none of the phage-resistant *L. monocytogenes* mutants exhibited resistance mechanisms involving post adsorption interference with phage replication cycle. However several of the mutants were identified as putative phase variants. This suggests that phase variation may appear a significant genetic mechanism for the pathogen survival under phage-mediated selective pressure. Mutant strains resistant to both phages had disruptive mutations in their rhamnose biosynthesis operon, when still possessing N-acetylglucosamine in their wall teichoic acid (WTA), whereas mutants resistant to only LP-125 lacked terminal N-acetylglucosamine in their WTA. According to Trudelle et al. [87], mutations conferring phage-resistance based on a loss of rhamnose biosynthesis capacity probably pose the greatest challenge for phage-based biocontrol in *L. monocytogenes* serotype 1/2a strains. Based on susceptibility tests to the diverse collection of the *Listeria* phages the authors found that these strains conferred resistance to almost all of the phages. However, one (out of 120) *Listeria* phage LP-018 was able to lyse a phage-resistant *L. monocytogenes* strain lacking rhamnose in its cell WTA.

Song et al. [47] performed further studies on LP-018 bacteriophage. From the infected pathogen, the group selected 10 distinct *L. monocytogenes* 10403S LP-018-insensitive mutants. The mutants had a single mutation in LMRG_00278 or LMRG_01613 or in both genes. The phage was still able to bind to a representative phage-insensitive mutant with mutations in both genes, what clearly suggest that these mutations confer resistance through a mechanism other than adsorption inhibition. According to the authors, these mutations appear to affect phages specifically in the *Homburgvirus* genus.

To determine if gaining phage resistance can be correlated with the development of resistance (or reduced susceptibility) to antimicrobials, European Food Safety Authority (EFSA) was requested to evaluate this possibility in strains of *Listeria* which have become resistant to P100 phage. *L. monocytogenes* P100-resistant mutants originating from 21 *Listeria* strains (20 *L. monocytogenes*, 1 *Listeria ivonovii*), all initially susceptible to P100, were provided to the EFSA panel. Most of the strains (out of 34) did not change their sensitivity to antimicrobials. There were, however, 4 exceptional mutant-strains. In two mutants, P100 resistance resulted in the development of full resistance to rifamycin, however mutants were derived from a single strain of *L. monocytogenes* with intermediate initial resistance to this antimicrobial. On the other hand, gaining P100 resistance resulted in an apparent reversion to sensitivity to the antimicrobials in two strains; one exhibiting initial resistance to ciprofloxacin and second strain exhibiting initial erythromycin resistance. The reasons behind these changes are currently unknown [88].

Recent findings also suggest that serovar 4b strains of *L. monocytogenes* can gain resistance to bacteriophages but at the cost of its virulence. Clones of the pathogen with mutations in genes conferring teichoic acid glycosylation and causing a loss of galactose from both WTA and lipoteichoic acid molecules, resulting in a switch from serovar 4b to 4d and simultaneously phage resistance. Surprisingly, the loss of the galactose moiety apart from preventing phage adsorption interfere with host cell invasion. It was shown that galactose decoration is required for efficient function of a major virulence factor mediating host cell entry [89].

Many mechanisms underlying phage resistance are still not known. Fister et al. [85] concluded that the application of bacteriophage P100 could be recommended as an additional measure in situations if accompanying actions are required. These actions should impede bacteriophage from spreading and allow the subsequent elimination of P100 from the production environment. According to the authors, the routine use of P100 cannot be supported. EFSA panel stated in their recommendations that if Listex™ P100 is authorized and used, the food business operators should, on a regular basis, examine the susceptibility to Listex™ P100 of *L. monocytogenes* strains isolated from the raw materials/processing plant and if resistant strains occur, consider corrective actions [88]. Admittedly, these two conclusions relate to phage P100, however, similar mechanisms apply also to any other phages, thus that conclusions can be extended to potential industrial utilization of all other *Listeria* bacteriophages treatments.

## 5. Legislation of *Listeria* Bacteriophages in Food Products

Despite many successful trials of using bacteriophages in food products, their usage is allowed only in some countries and regulations relate only to individual bacteriophage products.

Today, LISTEX™ P100 is used in the USA, Canada and Switzerland [12]. It is further accepted as a processing aid in Australia, New Zealand, Israel, Switzerland, Canada and others [21], including The Netherlands (EU) [12,21]. In the European Union however, LISTEX™ P100 is not on the QPS (qualified presumption of safety) list. In their latest opinion regarding this product, EFSA stated that concerning the toxicological safety of the substance the bacteriophage P100 is not considered to pose a risk to human health and no food safety concerns are foreseen regarding the use of the industrially produced ListexTM P100 preparation during the processing of RTE foods. However, their recommendations were to perform more studies on Listex™P100 efficacy. They also concluded that further studies on the mechanisms by which *L. monocytogenes* strains exhibiting resistance to certain therapeutic antimicrobials become sensitive to these antimicrobials following the development of resistance to P100 [88].

In the USA use of P100 (present in the PhageGuard LISTEX™ P100) has GRAS (Generally Recognized As Safe) status conferred by FDA (Food and Drug Administration). Product was first allowed in 2006 as antimicrobial to control *Listeria monocytogenes* in brie, cheddar, Swiss and other cheeses that are normally aged and ripened [90] and one year later range of use were extended to control of *Listeria monocytogenes* in foods in general, including meat and poultry products, at levels up to 10^9^ plaque-forming units per gram [91]. The mix of six bacteriophages LIST-36, LMSP-25, LMTA-34, LMTA-57, LMTA-94 and LMTA-148 (components of ListShield™) also gained GRAS status in 2014 for use as an antimicrobial to control *Listeria monocytogenes* in fish and shellfish, fresh and processed fruits, fresh and processed vegetables and dairy products applied to food surfaces up to 1 × 10^6^ plaque-forming units per gram of food [92].

## 6. Conclusions

*Listeria monocytogenes* is a pathogen with strict legal limitations in many countries, though outbreaks of listeriosis still occur on a regular basis. Phage-based inhibition of pathogens in food products is considered as an innovative method for improvement of food safety. Bacteriophages are proved to be effective for reducing *Listeria monocytogenes* in food products and on the contaminated surfaces under certain conditions, thereby they might be a useful tool for industrial applications.

Prediction of the behavior of phages in a complex food matrix is very difficult and the effectiveness of phages is largely defined by the food itself [39]. To overcome this problem there was an approach with the application of Artificial Neural Networks for prediction of *L. monocytogenes* growth with phage treated produce, which brought better results (a better relationship between the predicted and the actual data was enhanced) than in other models [65]. Even with such technologies available, empirical data are required and what Guenther et al. [39] suggested is still valid—the application must be optimized for individual food systems. Effectiveness of the treatment depends not only on the food itself but also on *L. monocytogenes* strains occurring in the product, because phage performance may vary depending on different pathogen strains. In addition, succeeding with inhibition of the pathogen depends on the initial contamination level. Predicting these two factors (a strain of the pathogen and its concentration) in real food processing is very hard if not impossible.

Many authors agreed that phages alone are not a solution for *Listeria monocytogenes* contamination in food for many reasons. However as bacteriophages’ effectiveness is significantly enhanced when used with other antilisterial factors, they may still be very beneficial both for food producers and for consumers.

Even though many facts are already known, further studies regarding phage-based *Listeria monocytogenes* inhibition in foods are needed. Especially studying performance, safety and properties of other promising bacteriophages in food product models would be beneficial, as the vast majority of such experiments to date were done only with PhageGuard Listex™ and ListShield™. As well as studies on complex mechanisms underlying combined treatment with phages and alternative antilisterial factors such as essential oils in varying food could also bring many interesting data. What is more, studies regarding phage resistance mechanisms, for example, as suggested by EFSA [88], studies on the mechanisms by which *L. monocytogenes* strains exhibiting resistance to certain therapeutic antimicrobials become sensitive to these antimicrobials following the development of resistance to P100 are also required, as well as monitoring of the occurrence of phage-resistant strains in food processing plants, where allowed phage products are used.

## Figures and Tables

**Table 1:** Summarized attempts of phage-based *Listeria monocytogenes* inhibition in food products.

**Phage(s)**	**Food Product(s)**	**Cofactor(s) Tested**	**References**
LH7	Vacuum packed beef	Nisin	[48]
ListShield™ ^1^ LM-103 ^2^	Red Delicious apple slices and honeydew melons slices	Nisin	[49]
ListShield™ ^1^	Golden Delicious apples and Honeydew melon pieces		[50]
PhageGuard Listex™ ^3^	Contaminated surface-ripened red-smear soft cheese (type “Munster”)		[28]
A511 P100 to a lesser extend	Various RTE food products: hot dogs (sausages), cooked and sliced turkey breast meat (cold cuts), smoked salmon, mixed seafood (cooked and chilled cocktail of shrimp, mussels and calamari), chocolate milk (pasteurized, 3.5% fat), mozzarella cheese brine (unsalted pasteurized whey from plastic bag containers containing fresh mozzarella cheese), iceberg lettuce (leaves), cabbage (sliced fresh leaves)		[39]
PhageGuard Listex™	Slices of cooked ham	Protective culture - *Lactobacillus sakei* TH1	[38]
PhageGuard Listex™	Channel catfish fillets		[44]
PhageGuard Listex™	Fresh raw salmon fillets		[51]
FWLLm1 ^4^	Commercially available RTE chicken breast roll which had been cooked in its packaging		[52]
PhageGuard Listex™	Brazilian sausage made with pork meat		[53]
A511	Soft ripened white mold and red-smear cheeses		[40]
PhageGuard Listex™	Queso fresco cheese	lauric arginate (LAE), potassium lactate–sodium diacetate mixture (PL–SD)	[42]
PhageGuard Listex™	RTE sliced roast beef and cooked turkey	potassium lactate (PL) (on the turkey), sodium diacetate (SD) and potassium lactate (on the roast beef)	[54]
PhageGuard Listex™	Cold-smoked salmon	Nisin lauric arginate	[55]
PhageGuard Listex™	Soft cheeses Minas Frescal and Coalho		[56]
PhageGuard Listex™	Fruit pieces/slices and juices made of: apples (*Malus domestica* cv. Golden Delicious), pears (*Pyrus communis* cv. Conference), melons (*Cucumis melo* L. var. ‘Piel de sapo’)		[57]
ListShield™	Honeydew melon slices	*Gluconobacter asaii*	[58]
ListShield™	Various RTE food products: Long-leaf green lettuce A hard, pasteurized cheese Cold smoked salmon Gala apple slices Frozen entrée samples (fully-cooked, prepackaged meals served in-flight on airplanes)		[22]
PhageGuard Listex™	Raw hake fillets, raw salmon fillets and smoked filleted salmon	enterocin AS-48	[59]
PhageGuard Listex™	Dry-cured ham slices		[60]
ListShield™	Romaine lettuce (biofilm on leaves)		[13]
Cocktail of: LinM-AG8 LmoM-AG13 LmoM-AG20 PhageGuard Listex™	Freshly cut cantaloupes (tested with phage cocktail) RTE cooked turkey (tested with P100)		[61]
LMP1 and LMP7	Pasteurized milk		[62]
PhageGuard Listex™	Sashimi–sliced raw tuna loins (*Thunnus albacares*)		[63]
PhageGuard Listex™	Pork ham slices	Nisin	[64]
ListShield™	Chicken breasts	UV-C light	[43]
PhageGuard Listex™	Tomato (*Lycopersicon esculentum*) and carrot (*Daucus carota* subsp. *sativus*)	Sucrose monolaurate	[65]
P100 isolated from PhageGuard Listex™	Coleslaw	Nisaplin ^®^ (nisin)	[66]
ListShield™	Cheese models		[67]
PhageGuard Listex™	Cut curly endive heads		[68]
PhageGuard Listex™	Ultra-high temperature (UHT) whole milk (3.6% fat content)	High pressure, pediocin PA-1	[69]
PhageGuard Listex™	Rakfisk–Norwegian fermented fish product (trout and char)		[70]

^1^ ListShield™ was formerly referred to as LMP-102. ^2^ mix of 14 distinct lytic phages by Intralytix, Inc. (Baltimore, Md.). ^3^ PhageGuard Listex™ was formerly referred to Listex™ or P100. ^4^ phage was isolated by authors from sheep feces.

**Table 2:** Summarized attempts of immobilizing antilisterial bacteriophages.

Phage(s)	Immobilized Material	Food Product	References
LinM-AG8 LmoM-AG13 LmoM-AG20	Cellulose membranes	Ready-to-eat, oven-roasted turkey breast	[75]
Cocktail of 3 phages: LinM-AG8 LmoM-AG13 LmoM-AG20 and PhageGuard Listex™ (formerly Listex™; P100)	Cellulose membranes	Freshly cut cantaloupes (tested with phage cocktail) RTE cooked turkey (tested with P100)	[61]
A511	Xanthan coatings on Poly(lactic acid) films	Precooked sliced turkey breast	[76]

**Table 3:** Summarized attempts of phage-based *Listeria monocytogenes* inhibition on different surfaces.

Phage(s)	Tested Material	References
H387 H387-A 2671	Stainless steel and polypropylene surfaces	[78]
Bred phage ATCC 23074-B1	Stainless steel	[79]
PhageGuard Listex™ ^1^	Stainless steel	[80]
PhageGuard Listex™ ^1^	Stainless steel	[83]
LiMN4L LiMN4p LiMN17	Stainless steel	[82]
PhageGuard Listex™ ^1^	Stainless steel	[77]
PhageGuard Listex™ ^1^	Stainless Steel	[60]
ListShield™ ^2^	Stainless steel and rubber surfaces	[13]
PhageGuard Listex™ ^1^	Polystyrene	[81]

^1^ PhageGuard Listex™ was formerly referred to Listex™ or P100.^2^ ListShield™ was formerly referred to as LMP-102.

## References

[1] Jeffs E., Williman J., Brunton C., Gullam J., Walls T. (2020). The epidemiology of listeriosis in pregnant women and children in New Zealand from 1997 to 2016: An observational study. BMC Public Health.

[2] Kaptchouang Tchatchouang C.-D., Fri J., De Santi M., Brandi G., Schiavano G.F., Amagliani G., Ateba C.N. (2020). Listeriosis Outbreak in South Africa: A Comparative Analysis with Previously Reported Cases Worldwide. Microorganisms.

[3] Kramarenko T., Roasto M., Meremäe K., Kuningas M., Põltsama P., Elias T. (2013). *Listeria monocytogenes* prevalence and serotype diversity in various foods. Food Control.

[4] Smith A.M., Tau N.P., Smouse S.L., Allam M., Ismail A., Ramalwa N.R., Disenyeng B., Ngomane M., Thomas J. (2019). Outbreak of *Listeria monocytogenes* in South Africa, 2017–2018: Laboratory Activities and Experiences Associated with Whole-Genome Sequencing Analysis of Isolates. Foodborne Pathog. Dis..

[5] Buchanan R.L., Gorris L.G.M., Hayman M.M., Jackson T.C., Whiting R.C. (2017). A review of *Listeria monocytogenes*: An update on outbreaks, virulence, dose-response, ecology and risk assessments. Food Control.

[6] Chen J.-Q., Regan P., Laksanalamai P., Healey S., Hu Z. (2017). Prevalence and methodologies for detection, characterization and subtyping of *Listeria monocytogenes* and *L. ivanovii* in foods and environmental sources. Food Sci. Hum. Wellness.

[7] Rebuffo-Scheer C.A., Schmitt J., Scherer S. (2007). Differentiation of *Listeria monocytogenes* Serovars by Using Artificial Neural Network Analysis of Fourier-Transformed Infrared Spectra. Appl. Environ. Microbiol..

[8] Feng Y., Yao H., Chen S., Sun X., Yin Y., Jiao X. (2020). Rapid Detection of Hypervirulent Serovar 4h *Listeria monocytogenes* by Multiplex PCR. Front. Microbiol..

[9] Jordan K., McAuliffe O. (2018). Listeria monocytogenes in Foods. Advances in Food and Nutrition Research.

[10] Kim J.-W., Siletzky R.M., Kathariou S. (2008). Host Ranges of *Listeria*-Specific Bacteriophages from the Turkey Processing Plant Environment in the United States. Appl. Environ. Microbiol..

[11] Radoshevich L., Cossart P. (2018). *Listeria monocytogenes*: Towards a complete picture of its physiology and pathogenesis. Nat. Rev. Microbiol..

[12] Aprea G., Zocchi L., Di Fabio M., De Santis S., Prencipe V.A., Migliorati G. (2018). The applications of bacteriophages and their lysins as biocontrol agents against the foodborne pathogens *Listeria monocytogenes* and *Campylobacter* spp.: An updated look. Vet. Ital..

[13] Sadekuzzaman M., Yang S., Mizan F.R., Kim H.-S., Ha S.-D. (2017). Effectiveness of a phage cocktail as a biocontrol agent against *L. monocytogenes* biofilms. Food Control.

[14] Gray J.A., Chandry P.S., Kaur M., Kocharunchitt C., Bowman J.P., Fox E.M. (2018). Novel Biocontrol Methods for *Listeria monocytogenes* Biofilms in Food Production Facilities. Front. Microbiol..

[15] Gutiérrez D., Rodríguez-Rubio L., Martínez B., Rodríguez A., García P. (2016). Bacteriophages as Weapons Against Bacterial Biofilms in the Food Industry. Front. Microbiol..

[16] Self J.L., Conrad A., Stroika S., Jackson A., Whitlock L., Jackson K.A., Beal J., Wellman A., Fatica M.K., Bidol S. (2019). Multistate Outbreak of Listeriosis Associated with Packaged Leafy Green Salads, United States and Canada, 2015–2016. Emerg. Infect. Dis..

[17] Paduro C., Montero D.A., Chamorro N., Carreño L.J., Vidal M., Vidal R. (2020). Ten years of molecular epidemiology surveillance of *Listeria monocytogenes* in Chile 2008–2017. Food Microbiol..

[18] Archer D.L. (2018). The evolution of FDA’s policy on *Listeria monocytogenes* in ready-to-eat foods in the United States. Curr. Opin. Food Sci..

[19] Jami M., Ghanbari M., Zunabovic M., Domig K.J., Kneifel W. (2014). *Listeria monocytogenes* in Aquatic Food Products-A Review. Compr. Rev. Food Sci. Food Saf..

[20] Batinovic S., Wassef F., Knowler S.A., Rice D.T., Stanton C.R., Rose J., Tucci J., Nittami T., Vinh A., Drummond G.R. (2019). Bacteriophages in Natural and Artificial Environments. Pathogens.

[21] Połaska M., Sokołowska B. (2019). Bacteriophages—A new hope or a huge problem in the food industry. AIMS Microbiol..

[22] Perera M.N., Abuladze T., Li M., Woolston J., Sulakvelidze A. (2015). Bacteriophage cocktail significantly reduces or eliminates *Listeria monocytogenes* contamination on lettuce, apples, cheese, smoked salmon and frozen foods. Food Microbiol..

[23] Żbikowska K., Michalczuk M., Dolka B. (2020). The Use of Bacteriophages in the Poultry Industry. Animals.

[24] Hagens S., Loessner M. (2010). Bacteriophage for Biocontrol of Foodborne Pathogens: Calculations and Considerations. Curr. Pharm. Biotechnol..

[25] Myelnikov D. (2018). An Alternative Cure: The Adoption and Survival of Bacteriophage Therapy in the USSR, 1922–1955. J. Hist. Med. Allied Sci..

[26] Żaczek M., Weber-Dąbrowska B., Międzybrodzki R., Łusiak-Szelachowska M., Górski A. (2020). Phage Therapy in Poland—A Centennial Journey to the First Ethically Approved Treatment Facility in Europe. Front. Microbiol..

[27] Gaynes R. (2017). The Discovery of Penicillin—New Insights After More Than 75 Years of Clinical Use. Emerg. Infect. Dis..

[28] Carlton R.M., Noordman W.H., Biswas B., de Meester E.D., Loessner M.J. (2005). Bacteriophage P100 for control of *Listeria monocytogenes* in foods: Genome sequence, bioinformatic analyses, oral toxicity study and application. Regul. Toxicol. Pharmacol..

[29] Hudson J.A., Billington C., Carey-Smith G., Greening G. (2005). Bacteriophages as Biocontrol Agents in Food. J. Food Prot..

[30] Vu H.T.K., Benjakul S., Vongkamjan K. (2019). Characterization of *Listeria* prophages in lysogenic isolates from foods and food processing environments. PLoS ONE.

[31] Jamal M., Bukhari S.M.A.U.S., Andleeb S., Ali M., Raza S., Nawaz M.A., Hussain T., Rahman S.U., Shah S.S.A. (2019). Bacteriophages: An overview of the control strategies against multiple bacterial infections in different fields. J. Basic Microbiol..

[32] Monk A.B., Rees C.D., Barrow P., Hagens S., Harper D.R. (2010). Bacteriophage applications: Where are we now?. Lett. Appl. Microbiol..

[33] Moye Z., Woolston J., Sulakvelidze A. (2018). Bacteriophage Applications for Food Production and Processing. Viruses.

[34] Schellekens M.M., Woutersi J., Hagens S., Hugenholtz J. (2007). Bacteriophage P100 application to control *Listeria monocytogenes* on smeared cheese. Milchwissenschaft.

[35] Hagens S., Loessner M.J. (2014). Phages of *Listeria* offer novel tools for diagnostics and biocontrol. Front. Microbiol..

[36] Klumpp J., Loessner M.J. (2013). *Listeria* phages: Genomes, evolution and application. Bacteriophage.

[37] Vongkamjan K., Roof S., Stasiewicz M.J., Wiedmann M. (2013). Persistent *Listeria monocytogenes* subtypes isolated from a smoked fish processing facility included both phage susceptible and resistant isolates. Food Microbiol..

[38] Holck A., Berg J. (2009). Inhibition of *Listeria monocytogenes* in Cooked Ham by Virulent Bacteriophages and Protective Cultures. Appl. Environ. Microbiol..

[39] Guenther S., Huwyler D., Richard S., Loessner M.J. (2009). Virulent Bacteriophage for Efficient Biocontrol of *Listeria monocytogenes* in Ready-To-Eat Foods. Appl. Environ. Microbiol..

[40] Guenther S., Loessner M.J. (2011). Bacteriophage biocontrol of *Listeria monocytogenes* on soft ripened white mold and red-smear cheeses. Bacteriophage.

[41] Schmelcher M., Loessner M.J. (2016). Bacteriophage endolysins: Applications for food safety. Curr. Opin. Biotechnol..

[42] Soni K.A., Desai M., Oladunjoye A., Skrobot F., Nannapaneni R. (2012). Reduction of *Listeria monocytogenes* in queso fresco cheese by a combination of listericidal and listeriostatic GRAS antimicrobials. Int. J. Food Microbiol..

[43] Yang S., Sadekuzzaman M., Ha S.-D. (2017). Reduction of *Listeria monocytogenes* on chicken breasts by combined treatment with UV-C light and bacteriophage ListShield. LWT.

[44] Soni K.A., Nannapaneni R., Hagens S. (2010). Reduction of *Listeria monocytogenes* on the Surface of Fresh Channel Catfish Fillets by Bacteriophage Listex P100. Foodborne Pathog. Dis..

[45] Ahmadi H., Radford D., Kropinski A.M., Lim L.-T., Balamurugan S. (2017). Thermal-Stability and Reconstitution Ability of *Listeria* Phages P100 and A511. Front. Microbiol..

[46] Schmuki M.M., Erne D., Loessner M.J., Klumpp J. (2012). Bacteriophage P70: Unique Morphology and Unrelatedness to Other *Listeria* Bacteriophages. J. Virol..

[47] Song Y., Peters T.L., Bryan D.W., Hudson L.K., Denes T.G. (2019). Homburgvirus LP-018 Has a Unique Ability to Infect Phage-Resistant *Listeria monocytogenes*. Viruses.

[48] Dykes G.A., Moorhead S.M. (2002). Combined antimicrobial effect of nisin and a listeriophage against *Listeria monocytogenes* in broth but not in buffer or on raw beef. Int. J. Food Microbiol..

[49] Leverentz B., Conway W.S., Camp M.J., Janisiewicz W.J., Abuladze T., Yang M., Saftner R., Sulakvelidze A. (2003). Biocontrol of *Listeria monocytogenes* on Fresh-Cut Produce by Treatment with Lytic Bacteriophages and a Bacteriocin. Appl. Environ. Microbiol..

[50] Leverentz B., Conway W.S., Janisiewicz W., Camp M.J. (2004). Optimizing Concentration and Timing of a Phage Spray Application To Reduce *Listeria monocytogenes* on Honeydew Melon Tissue. J. Food Prot..

[51] Soni K.A., Nannapaneni R. (2010). Bacteriophage Significantly Reduces *Listeria monocytogenes* on Raw Salmon Fillet Tissue. J. Food Prot..

[52] Bigot B., Lee W.-J., McIntyre L., Wilson T., Hudson J.A., Billington C., Heinemann J.A. (2011). Control of *Listeria monocytogenes* growth in a ready-to-eat poultry product using a bacteriophage. Food Microbiol..

[53] Rossi L.P.R., Almeida R.C.C., Lopes L.S., Figueiredo A.C.L., Ramos M.P.P., Almeida P.F. (2011). Occurrence of *Listeria* spp. in Brazilian fresh sausage and control of *Listeria monocytogenes* using bacteriophage P100. Food Control.

[54] Chibeu A., Agius L., Gao A., Sabour P.M., Kropinski A.M., Balamurugan S. (2013). Efficacy of bacteriophage LISTEX^TM^P100 combined with chemical antimicrobials in reducing *Listeria monocytogenes* in cooked turkey and roast beef. Int. J. Food Microbiol..

[55] Soni K.A., Shen Q., Nannapaneni R. (2014). Reduction of *Listeria monocytogenes* in cold-smoked salmon by bacteriophage P100, nisin and lauric arginate, singly or in combinations. Int. J. Food Sci. Technol..

[56] Silva E.N.G., Figueiredo A.C.L., Miranda F.A., de Castro Almeida R.C. (2014). Control of *Listeria monocytogenes* growth in soft cheeses by bacteriophage P100. Braz. J. Microbiol..

[57] Oliveira M., Viñas I., Colàs P., Anguera M., Usall J., Abadias M. (2014). Effectiveness of a bacteriophage in reducing *Listeria monocytogenes* on fresh-cut fruits and fruit juices. Food Microbiol..

[58] Hong Y.-P. (2015). Combining of Bacteriophage and *G. asaii* Application to Reduce *L. monocytogenes* on Fresh-Cut Melon under Low Temperature and Packing with Functional Film. J. Food Nutr. Sci..

[59] Baños A., García-López J.D., Núñez C., Martínez-Bueno M., Maqueda M., Valdivia E. (2016). Biocontrol of *Listeria monocytogenes* in fish by enterocin AS-48 and *Listeria* lytic bacteriophage P100. LWT-Food Sci. Technol..

[60] Iacumin L., Manzano M., Comi G. (2016). Phage Inactivation of *Listeria monocytogenes* on San Daniele Dry-Cured Ham and Elimination of Biofilms from Equipment and Working Environments. Microorganisms.

[61] Lone A., Anany H., Hakeem M., Aguis L., Avdjian A.-C., Bouget M., Atashi A., Brovko L., Rochefort D., Griffiths M.W. (2016). Development of prototypes of bioactive packaging materials based on immobilized bacteriophages for control of growth of bacterial pathogens in foods. Int. J. Food Microbiol..

[62] Lee S., Kim M.G., Lee H.S., Heo S., Kwon M., Kim G. (2017). Isolation and Characterization of *Listeria* phages for Control of Growth of *Listeria monocytogenes* in Milk. Korean J. Food Sci. Anim. Resour..

[63] Miguéis S., Saraiva C., Esteves A. (2017). Efficacy of LISTEX P100 at Different Concentrations for Reduction of *Listeria monocytogenes* Inoculated in Sashimi. J. Food Prot..

[64] Figueiredo A.C.L., Almeida R.C.C. (2017). Antibacterial efficacy of nisin, bacteriophage P100 and sodium lactate against *Listeria monocytogenes* in ready-to-eat sliced pork ham. Braz. J. Microbiol..

[65] Oladunjoye A.O., Oyewole S.A., Singh S., Ijabadeniyi O.A. (2017). Prediction of *Listeria monocytogenes* ATCC 7644 growth on fresh-cut produce treated with bacteriophage and sucrose monolaurate by using artificial neural network. LWT-Food Sci. Technol..

[66] Lewis R., Bolocan A.S., Draper L.A., Ross R.P., Hill C. (2019). The Effect of a Commercially Available Bacteriophage and Bacteriocin on *Listeria monocytogenes* in Coleslaw. Viruses.

[67] Henderson L.O., Cabrera-Villamizar L.A., Skeens J., Kent D., Murphy S., Wiedmann M., Guariglia-Oropeza V. (2019). Environmental conditions and serotype affect *Listeria monocytogenes* susceptibility to phage treatment in a laboratory cheese model. J. Dairy Sci..

[68] Truchado P., Elsser-Gravesen A., Gil M.I., Allende A. (2020). Post-process treatments are effective strategies to reduce *Listeria monocytogenes* on the surface of leafy greens: A pilot study. Int. J. Food Microbiol..

[69] Komora N., Maciel C., Pinto C.A., Ferreira V., Brandão T.R.S., Saraiva J.M.A., Castro S.M., Teixeira P. (2020). Non-thermal approach to *Listeria monocytogenes* inactivation in milk: The combined effect of high pressure, pediocin PA-1 and bacteriophage P100. Food Microbiol..

[70] Axelsson L., Bjerke G.A., McLeod A., Berget I., Holck A.L. (2020). Growth Behavior of *Listeria monocytogenes* in a Traditional Norwegian Fermented Fish Product (Rakfisk) and Its Inhibition through Bacteriophage Addition. Foods.

[71] Fister S., Robben C., Witte A.K., Schoder D., Wagner M., Rossmanith P. (2016). Influence of Environmental Factors on Phage–Bacteria Interaction and on the Efficacy and Infectivity of Phage P100. Front. Microbiol..

[72] Tokman J.I., Kent D.J., Wiedmann M., Denes T. (2016). Temperature Significantly Affects the Plaquing and Adsorption Efficiencies of *Listeria* Phages. Front. Microbiol..

[73] Kim J.-W., Dutta V., Elhanafi D., Lee S., Osborne J.A., Kathariou S. (2012). A Novel Restriction-Modification System Is Responsible for Temperature-Dependent Phage Resistance in *Listeria monocytogenes* ECII. Appl. Environ. Microbiol..

[74] Chibeu A., Balamurugan S., Clokie M.R.J., Kropinski A.M., Lavigne R. (2018). Application of a Virucidal Agent to Avoid Overestimation of Phage Kill During Phage Decontamination Assays on Ready-to-Eat Meats. Bacteriophages.

[75] Anany H., Chen W., Pelton R., Griffiths M.W. (2011). Biocontrol of *Listeria monocytogenes* and Escherichia coli O157:H7 in Meat by Using Phages Immobilized on Modified Cellulose Membranes. Appl. Environ. Microbiol..

[76] Radford D., Guild B., Strange P., Ahmed R., Lim L.-T., Balamurugan S. (2017). Characterization of antimicrobial properties of Salmonella phage Felix O1 and *Listeria* phage A511 embedded in xanthan coatings on Poly(lactic acid) films. Food Microbiol..

[77] Chaitiemwong N., Hazeleger W.C., Beumer R.R. (2014). Inactivation of *Listeria monocytogenes* by Disinfectants and Bacteriophages in Suspension and Stainless Steel Carrier Tests. J. Food Prot..

[78] Roy B., Ackermann H.W., Pandian S., Picard G., Goulet J. (1993). Biological inactivation of adhering *Listeria monocytogenes* by *Listeria*phages and a quaternary ammonium compound. Appl. Environ. Microbiol..

[79] Hibma A. (1997). Infection and removal of L-forms of *Listeria monocytogenes* with bred bacteriophage. Int. J. Food Microbiol..

[80] Soni K.A., Nannapaneni R. (2010). Removal of *Listeria monocytogenes* Biofilms with Bacteriophage P100. J. Food Prot..

[81] Rodríguez-Melcón C., Capita R., García-Fernández C., Alonso-Calleja C. (2018). Effects of Bacteriophage P100 at Different Concentrations on the Structural Parameters of *Listeria monocytogenes* Biofilms. J. Food Prot..

[82] Ganegama Arachchi G.J., Cridge A.G., Dias-Wanigasekera B.M., Cruz C.D., McIntyre L., Liu R., Flint S.H., Mutukumira A.N. (2013). Effectiveness of phages in the decontamination of *Listeria monocytogenes* adhered to clean stainless steel, stainless steel coated with fish protein and as a biofilm. J. Ind. Microbiol. Biotechnol..

[83] Montañez-Izquierdo V.Y., Salas-Vázquez D.I., Rodríguez-Jerez J.J. (2012). Use of epifluorescence microscopy to assess the effectiveness of phage P100 in controlling *Listeria monocytogenes* biofilms on stainless steel surfaces. Food Control.

[84] Vongkamjan K., Benjakul S., Kim Vu H.T., Vuddhakul V. (2017). Longitudinal monitoring of *Listeria monocytogenes* and *Listeria* phages in seafood processing environments in Thailand. Food Microbiol..

[85] Fister S., Fuchs S., Stessl B., Schoder D., Wagner M., Rossmanith P. (2016). Screening and characterisation of bacteriophage P100 insensitive *Listeria monocytogenes* isolates in Austrian dairy plants. Food Control.

[86] Denes T., den Bakker H.C., Tokman J.I., Guldimann C., Wiedmann M. (2015). Selection and Characterization of Phage-Resistant Mutant Strains of *Listeria monocytogenes* Reveal Host Genes Linked to Phage Adsorption. Appl. Environ. Microbiol..

[87] Trudelle D.M., Bryan D.W., Hudson L.K., Denes T.G. (2019). Cross-resistance to phage infection in *Listeria monocytogenes* serotype 1/2a mutants. Food Microbiol..

[88] EFSA Panel on Biological Hazards (BIOHAZ) (2016). Evaluation of the safety and efficacy of Listex^TM^ P100 for reduction of pathogens on different ready-to-eat (RTE) food products. EFSA J..

[89] Sumrall E.T., Shen Y., Keller A.P., Rismondo J., Pavlou M., Eugster M.R., Boulos S., Disson O., Thouvenot P., Kilcher S. (2019). Phage resistance at the cost of virulence: *Listeria monocytogenes* serovar 4b requires galactosylated teichoic acids for InlB-mediated invasion. PLoS Pathog..

[90] U.S. Food & Drug Administration GRAS Notice No. 198. https://www.cfsanappsexternal.fda.gov/scripts/fdcc/index.cfm?set=GRASNotices&id=198.

[91] U.S. Food & Drug Administration GRAS Notice No. 218. https://www.cfsanappsexternal.fda.gov/scripts/fdcc/index.cfm?set=GRASNotices&id=218.

[92] U.S. Food & Drug Administration GRAS Notice No. 528. https://www.cfsanappsexternal.fda.gov/scripts/fdcc/index.cfm?set=GRASNotices&id=528.

